# Augmenting the Activity of Monoterpenoid Phenols against Fungal Pathogens Using 2-Hydroxy-4-methoxybenzaldehyde that Target Cell Wall Integrity

**DOI:** 10.3390/ijms161125988

**Published:** 2015-11-10

**Authors:** Jong H. Kim, Kathleen L. Chan, Noreen Mahoney

**Affiliations:** Foodborne Toxin Detection and Prevention Research Unit, Western Regional Research Center, Agricultural Research Service, United States Department of Agriculture, Albany, CA 94710, USA; kathy.chan@ars.usda.gov (K.L.C.); noreen.mahoney@ars.usda.gov (N.M.)

**Keywords:** antimycotic, benzaldehydes, cell wall integrity, chemosensitization, filamentous fungi, monoterpenoids, mycotoxins, *Saccharomyces cerevisiae*, signal transduction, synergism

## Abstract

Disruption of cell wall integrity system should be an effective strategy for control of fungal pathogens. To augment the cell wall disruption efficacy of monoterpenoid phenols (carvacrol, thymol), antimycotic potency of benzaldehyde derivatives that can serve as chemosensitizing agents were evaluated against strains of *Saccharomyces cerevisiae* wild type (WT), *slt2*Δ and *bck1*Δ (mutants of the mitogen-activated protein kinase (MAPK) and MAPK kinase kinase, respectively, in the cell wall integrity pathway). Among fourteen compounds investigated, *slt2*Δ and *bck1*Δ showed higher susceptibility to nine benzaldehydes, compared to WT. Differential antimycotic activity of screened compounds indicated “structure-activity relationship” for targeting the cell wall integrity, where 2-hydroxy-4-methoxybenzaldehyde (2H4M) exhibited the highest antimycotic potency. The efficacy of 2H4M as an effective chemosensitizer to monoterpenoid phenols (*viz*., 2H4M + carvacrol or thymol) was assessed in yeasts or filamentous fungi (*Aspergillus*, *Penicillium*) according to European Committee on Antimicrobial Susceptibility Testing or Clinical Laboratory Standards Institute M38-A protocols, respectively. Synergistic chemosensitization greatly lowers minimum inhibitory or fungicidal concentrations of the co-administered compounds. 2H4M also overcame the tolerance of two MAPK mutants (*sakA*Δ, *mpkC*Δ) of *Aspergillus fumigatus* to fludioxonil (phenylpyrrole fungicide). Collectively, 2H4M possesses chemosensitizing capability to magnify the efficacy of monoterpenoid phenols, which improves target-based (*viz*., cell wall disruption) antifungal intervention.

## 1. Introduction

Filamentous fungi in the genus *Aspergillus* are ubiquitous opportunistic pathogens, which are most notable as causative agents of highly enervating human diseases such as aspergillosis [1]. They form extremely invasive human infections, particularly in immunocompromised patients or in people suffering chronic granulomatosis [2,3,4]. *Aspergillus flavus* and *Aspergillus parasiticus* also produce highly (hepato)carcinogenic aflatoxins, which contaminate various agricultural/food commodities [5]. Filamentous fungi in the genus *Penicillium* also frequently cause food contamination or postharvest decay, where *P. expansum* is the main producer of the mycotoxin patulin that negatively affects human and animal health [6].

Mycotic diseases/infections are becoming a serious problem since effective antifungal drugs or fungicides, especially agents for treating drug/fungicide-resistant fungi, are very limited. Development of fungal resistance to conventional antimycotic agents not only triggers global public health issues, but also threatens the safety of food supplies, especially for the products susceptible to mycotoxin contamination [7,8]. For instance, continuous applications of widely used fungicides, such as strobilurins, fludioxonils, *etc.*, to agricultural fields resulted in the development of fungal resistance to (and thus, escape from) the toxicities of fungicides. Moreover, applying fungicides at suboptimal concentrations or time-points of fungal growth can potentiate toxin production by mycotoxigenic fungi [9,10]. Fungicide-potentiation of mycotoxin production in fungi, especially those resistant to fungicides, has been reported in various aflatoxin-, trichothecene-, citrinin-, and patulin-producing fungal pathogens (See Table 1). Accordingly, there is an urgent demand to increase the efficacy of conventional antimycotic drugs/fungicides or develop new intervention strategies, which can secure the safe production of crops and food as well as public health.

**Table 1:** Fungicide potentiation of mycotoxin production in fungal pathogens.

Fungi	Fungicide	Key Features (Potentiation of Mycotoxin Production)
*Aspergillus parasiticus*	Anilinopyrimidine	Correlation between fitness parameters and aflatoxigenicity [11]
*A. parasiticus*	Flusilazole	Highly aflatoxigenic, sterol demethylation inhibition-resistant isolates [12]
*A. parasiticus*	Phenylpyrrole	Highly aflatoxigenic, phenylpyrrole resistant isolates [13]
*Fusarium graminearum*	Carbendazim	Increased trichothecene production with carbendazim resistance [14]
*Fusarium* sp.	Strobilurins	Increased deoxynivalenol production by sub-optimal application of strobilurin [9]
*F. sporotrichioides*	Carbendazim	Higher mycotoxin production (T-2 toxin, 4,15-diacetoxyscirpenol, neosolaniol) with carbendazim resistance [15]
*Penicillium expansum*	Tebuconazole, Fludioxonil, *etc.*	Adverse effect of fitness penalties on the mycotoxigenicity of resistant isolates [16]
*P. expansum*	Benzimidazole	Highly mycotoxigenic field isolates resistant to the benzimidazoles [17]
*P. verrucosum*	Iprodione	Strong induction of mycotoxin biosynthesis by iprodione [18]

The cell wall integrity system of fungi is an effective target for control of fungal pathogens [19]. The cell wall integrity pathway is well elucidated and described in the model fungus *Saccharomyces cerevisiae*, where the operation of mitogen-activated protein kinase (MAPK) signaling pathway (*viz*., cell wall integrity pathway) is controlled by protein kinase C [20]. The *BCK1* and *SLT2* genes, which encode MAPK kinase kinase (MAPKKK) and MAPK, respectively (Table 2), in the pathway play crucial roles for maintaining cell wall integrity in fungi [20]. Studies have shown that genes in the cell wall integrity system in fungi, such as species in the genus *Aspergillus* and *S. cerevisiae*, are functionally well conserved [21,22]. Therefore, cell wall targeting drugs (e.g., echinocandins) could be administered as broad-spectrum antimycotic agents for control of filamentous or yeast fungal pathogens, including species of *Candida* ([23] and references therein).

Nevertheless, despite their usefulness as cell wall targeting drugs, echinocandins generally do not completely inhibit fungal growth ([24] and references therein), where the determination of precise endpoints for pathogen intervention is very rigorous [25]. Echinocandin treatment can also trigger a compensatory stimulation of chitin synthesis, which causes the development of fungal resistance to the drugs ([24] and references therein). Accordingly, development of new drugs or intervention strategies is continually required for effective control of fungal pathogens, especially the strains exhibiting drug or fungicide resistance.

**Table 2:** Microbial strains used in this study.

*Aspergillus*	Characteristics	Source/References
*A. flavus* 3357	Plant pathogen (aflatoxin), Human pathogen (aspergillosis), Reference aflatoxigenic strain used for genome sequencing	NRRL ^a^ [26]
*A. flavus* 4212	Plant pathogen (aflatoxin), Human pathogen (aspergillosis)	NRRL
*A. parasiticus* 5862	Plant pathogen (aflatoxin)	NRRL
*A. parasiticus* 2999	Plant pathogen (aflatoxin)	NRRL
*A. fumigatus* AF293	Human pathogen (aspergillosis), Parental strain, Reference clinical strain used for genome sequencing	[26,27]
*A. fumigatus sakA*Δ	Human pathogen (aspergillosis), Mitogen-Activated Protein Kinase (MAPK) gene deletion mutant derived from AF293	[27]
*A. fumigatus mpkC*Δ	Human pathogen (aspergillosis), Mitogen-Activated Protein Kinase (MAPK) gene deletion mutant derived from AF293	[28]
*P. expansum* W1	Plant pathogen (patulin), Parental strain	[29]
*P. expansum* FR2	Plant pathogen (patulin), Fludioxonil resistant mutant derived from *P. expansum* W1	[29]
*P. expansum* W2	Plant pathogen (patulin), Parental strain	[29]
*P. expansum* FR3	Plant pathogen (patulin), Fludioxonil resistant mutant derived from *P. expansum* W2	[29]
***Saccharomyces***	**Characteristics**	**Source/References**
*S. cerevisiae* BY4741	Model yeast, Parental strain (*Mat* a *his3*Δ*1* *leu2*Δ*0* *met15*Δ*0* *ura3*Δ*0*)	[30]
*S. cerevisiae slt2*Δ	MAPK mutant in cell wall integrity system derived from BY4741	[30]
*S. cerevisiae bck1*Δ	MAPK kinase kinase (MAPKKK) mutant derived from BY4741	[30]

^a^ NRRL, National Center for Agricultural Utilization Research, USDA-ARS, Peoria, IL, USA.

Antifungal chemosensitization is an intervention scheme for effective control of pathogenic fungi, where co-application of a selected natural or synthetic compound (*viz.*, a chemosensitizer or a chemosensitizing agent) with a conventional antifungal drug can intensify the drug efficacy [31]. Chemosensitization strategy makes the fungal pathogens highly susceptible to the drug co-administered, where the chemosensitizer significantly impaired fungal defense to the conventional drug. By definition, comparing to the traditional combination therapy (*viz.*, combined application of two or more commercial drugs), a chemosensitizer itself does not have to exhibit a high extent of antifungal potency. However, chemosensitization not only magnifies the efficacy of the antimycotic drug co-applied, but also overcomes pathogen resistance to conventional drugs [31]. For example, co-application of piperazinyl quinolone with the azole drug fluconazole (FLC) resulted in overcoming FLC resistance of *C. albicans*, while the compound showed no antimycotic activity when applied alone [32]. Also, co-administration of cyclobutene-dione (squarile) derivatives with FLC elevated the drug activity during the treatment of *C. albicans*, where the chemosensitizer(s) modulated the major facilitator superfamily transporter (Mdr1p; responsible for FLC resistance) of the pathogen [33]. Chemosensitization mediated by a d-octapeptide derivative further overcame FLC resistance in *S. cerevisiae* and pathogenic fungi [34]. Collectively, fungal intervention via chemosensitization could be an alternative to (or complement) current antifungal practices, for example, combination therapy.

Natural products that present no significant medical or environmental side effects are potential sources of antimycotic or antimycotoxigenic agents, either in their nascent structure or as leads for more potent derivatives [35]. For instance, benzo derivatives (such as vanillic or caffeic acid) not only inhibited the growth of filamentous fungal pathogens*,* but also disrupted the production of mycotoxins [36]. The redox-active natural products, such as phenolic agents, can be potent redox cyclers that prevent fungal growth by interfering cellular redox homeostasis (thus, triggering fungal oxidative stress) or by disrupting the integrity of cellular components [37,38]. For defense, the fungal antioxidant system or cell wall/membrane integrity system play important roles for fungal tolerance to the phenolic agents administered [37,38]. 

**Figure 1:**
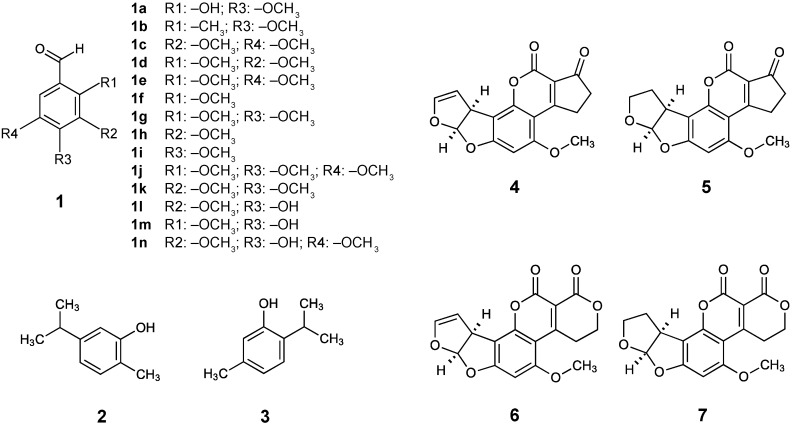
Structures of benzo derivatives, carvacrol, thymol, and aflatoxins used/detected in this study. (**1**) Benzaldehyde and derivatives: (**1a**) 2-Hydroxy-4-methoxybenzaldehyde, (**1b**) 2-Methyl-4-methoxybenzaldehyde, (**1c**) 3,5-Dimethoxybenzaldehyde, (**1d**) 2,3-Dimethoxybenzaldehyde, (**1e**) 2,5-Dimethoxybenzaldehyde, (**1f**) 2-Methoxybenzaldehyde, (**1g**) 2,4-Dimethoxybenzaldehyde, (**1h**) 3-Methoxybenzaldehyde, (**1i**) 4-Methoxybenzaldehyde, (**1j**) 2,4,5-Trimethoxybenzaldehyde, (**1k**) 3,4-Dimethoxybenzaldehyde, (**1l**) 4-Hydroxy-3-methoxybenzaldehyde, (**1m**) 4-Hydroxy-2-methoxybenzaldehyde, (**1n**) 3,5-Dimethoxy-4-hydroxybenzaldehyde; (**2**) Carvacrol (**5-Isopropyl-2-methylphenol**); (**3**) Thymol (**2-Isopropyl-5-methylphenol**); (**4**) Aflatoxin B_1_; (**5**) Aflatoxin B_2_; (**6**) Aflatoxin G_1_; (**7**) Aflatoxin G_2_.

Terpenoid phenols, such as carvacrol (**5-isopropyl-2-methylphenol**) and its structural isomer thymol (**2-isopropyl-5-methylphenol**) (Figure 1), have been demonstrated to be effective natural antimycotic agents by inhibiting the growth or activity of planktonic or biofilms of fungal pathogens ([39] and references therein). Carvacrol and thymol are generally regarded as safe (GRAS) reagents [40], and thus, are currently used as food additives. Genome-wide transcription profiling (microarray) study in the model fungus *S. cerevisiae* disclosed that genes in metabolic (energy, pyrimidine), biosynthetic, stress responses (oxidative, heat shock), *etc.*, were highly up- or down-regulated with the treatment of carvacrol, where the ion homeostasis mutant (*vma*Δ) was also hypersensitive to carvacrol treatment [39]. Similar microarray analysis in *S. cerevisiae* treated with thymol also revealed that genes involved in metabolism (sulfur, protein, thiamin, nucleic acid, *etc.*), mitochondrial function, organellar ribosome, cell proliferation, *etc.*, were up- or down-regulated by thymol application [41]. However, effects of carvacrol or thymol on the function of cell wall integrity system were undetermined in these studies.

The benzo derivative 2-hydroxy-4-methoxybenzaldehyde (2H4M) (Figure 1) is also a GRAS reagent [40], and hence, is currently used as a food additive. The 2H4M has been isolated from different plants as a natural compound, where it functioned as an insect repellent to protect food sources, *etc.* [42,43]. In this study, two *S. cerevisiae* cell wall integrity mutants (*bck1*Δ, *slt2*Δ), where genes in cell wall integrity MAPK pathway were deleted (Table 2), were examined to evaluate the efficacy of targeting cell wall integrity via natural product-based antifungal chemosensitization (namely, monoterpenoid phenols + 2H4M). The same intervention strategy was also investigated in filamentous fungal pathogens, such as *Aspergillus* and *Penicillium*, according to the Clinical Laboratory Standards Institute (CLSI) M38-A [44]. Results showed that: (1) 2H4M functioned as an effective antifungal chemosensitizer to augment the potency of monoterpenoid phenols; and (2) 2H4M overcame the tolerance of *A. fumigatus* MAPK mutants (*sakA*Δ, *mpkC*Δ) to fludioxonil, a phenylpyrrole fungicide.

## 2. Results and Discussion

### 2.1. Identification of 2-Hydroxy-4-methoxybenzaldehyde (2H4M) as the Most Potent Antifungal Benzaldehyde Analog via Yeast Screening: Structure-Activity Relationship

Antifungal efficacy of fourteen analogs of benzaldehyde (BA; basic structure) was investigated against the wild type (WT) and two cell wall integrity mutants (*bck1*Δ, *slt2*Δ) of *S. cerevisiae*, in *in vitro* agar plate (yeast dilution) bioassays. The *bck1*Δ and *slt2*Δ previously showed hypersensitivity to cell wall perturbing agents, such as caspofungin [45], and hence, can serve as screening tools for identifying new cell wall disrupting agents. Firstly, 2H4M was found to possess the highest antifungal activity against *S. cervisiae* strains (*viz.*, no growth of *bck1*Δ, *slt2*Δ and WT at 5.0 mM cutoff) (Table 3). Test benzaldehydes were further classified into three groups based on the level of antifungal potency against *bck1*Δ and *slt2*Δ mutants as follows: Group 1, 2H4M, 2-methyl-4-methoxybenzaldehyde, 3,5-dimethoxybenzaldehyde (3,5-DMBA), 2,3-dimethoxybenzaldehyde (2,3-DMBA) (Complete growth inhibition of *bck1*Δ and *slt2*Δ mutants at 5.0 mM cutoff (*viz*., growth score = 0; See Experimental section)); Group 2, 2,5-dimethoxybenzaldehyde (2,5-DMBA), 2-methoxybenzaldehyde, 2,4-dimethoxybenzaldehyde (2,4-DMBA), 3-methoxybenzaldehyde, 4-methoxybenzaldehyde [Moderate growth inhibition of *bck1*Δ and *slt2*Δ mutants at 5.0 mM cutoff (*viz*., growth score = 1 to 4)]; and Group 3, 2,4,5-trimethoxybenzaldehyde, 3,4-dimethoxybenzaldehyde (3,4-DMBA), 4-hydroxy-3-methoxybenzaldehyde, 4-hydroxy-2-methoxybenzaldehyde (4H2M), 3,5-dimethoxy-4-hydroxybenzaldehyde, BA (No growth inhibition of *bck1*Δ and *slt2*Δ mutants at 5.0 mM cutoff (*viz*., growth score = 6)) (Table 3).

**Table 3:** Growth scores of yeasts at 5 mM (cutoff) of benzaldehyde derivatives during yeast dilution bioassay (0, No growth; 6, Full growth; See Experimental section).

Benzaldehyde Derivatives	WT	*slt2*Δ	*bck1*Δ
2-Hydroxy-4-methoxybenzaldehyde	0	0	0
2-Methyl-4-methoxybenzaldehyde	1	0	0
3,5-Dimethoxybenzaldehyde	1	0	0
2,3-Dimethoxybenzaldehyde	2	0	0
2,5-Dimethoxybenzaldehyde	2	1	1
2-Methoxybenzaldehyde	3	1	1
2,4-Dimethoxybenzaldehyde	4	3	3
3-Methoxybenzaldehyde	6	3	2
4-Methoxybenzaldehyde	6	4	4
2,4,5-Trimethoxybenzaldehyde	6	6	6
3,4-Dimethoxybenzaldehyde	6	6	6
4-Hydroxy-3-methoxybenzaldehyde	6	6	6
4-Hydroxy-2-methoxybenzaldehyde	6	6	6
3,5-Dimethoxy-4-hydroxybenzaldehyde	6	6	6
Benzaldehyde	6	6	6

**Figure 2:**
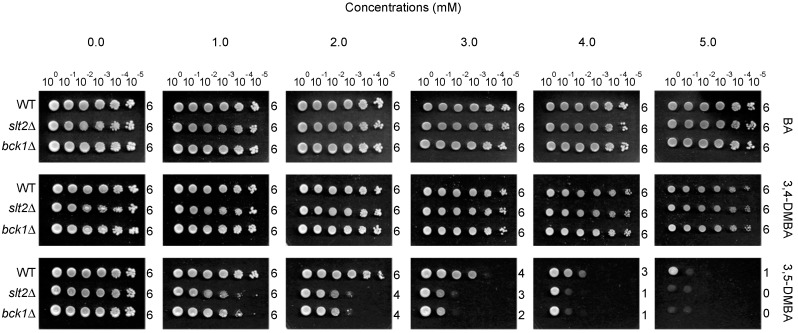
Differential susceptibility of *S. cerevisiae* strains to benzaldehyde derivatives. Exemplary yeast dilution bioassay showed that *S. cerevisiae* cell wall integrity mutants (*slt2*Δ, *bck1*Δ) were more susceptible to 3,5-DMBA compared to WT. Results also showed the structure-activity relationship, where 3,5-DMBA possessed potent antifungal activity (at 1 to 5 mM), while BA and 3,4-DMBA exhibited no antimycotic potency at the same concentrations. Numbers on right side of each row (0 to 6) indicate growth scores (See Experimental section).

Structure-activity relationships were also found with the test compounds. Structural-activity relationship in this study is defined as the relationship between the structures of test compounds (benzaldehydes), *i.e.*, kinds of chemical groups/side chains and/or their position on the benzene ring, and the level of antifungal activity. For example, the growth of *S. cerevisiae bck1*Δ and *slt2*Δ (and WT) was almost not affected by 3,4-DMBA (growth score = 6), while that of test strains treated with 3,5-DMBA (having a shift of a methoxyl residue from #4 to #5 position on the benzene ring) was greatly disrupted, where the level of growth inhibition was commensurate with compound concentration (1.0 to 5.0 mM) (*viz*., growth score = 0 to 5) (Figure 2; Table 3). Of note is that, while 2H4M was the most potent antifungal compound tested against WT, *bck1*Δ and *slt2*Δ (growth score = 0 at 5.0 mM cutoff; Table 3), the 4H2M, where the hydroxyl- and methoxyl-residues were reciprocally exchanged compared to 2H4M, exhibited almost no antimycotic activity in the same yeast strains (growth score = 6 at 5.0 mM cutoff; Table 3). Besides, the 2,3- and 2,5-DMBA exhibited higher antifungal potency (against the *bck1*Δ and *slt2*Δ) compared to 2,4-DMBA [order of activity (high to low): 2,3-DMBA (growth score = 0) > 2,5-DMBA (growth score = 1) > 2,4-DMBA (growth score = 6)]. Similar characteristics could be found in a prior study with quinone derivatives, where activities or functions of enzymes or cellular proteins were disrupted mainly by those analogs possessing an *ortho*- or *para*-quinonoid structure [46].

Collectively, nine out of fifteen benzaldehydes (including BA as the basic structure) negatively affected the growth of *bck1*Δ and *slt2*Δ, the cell wall integrity mutants of *S. cerevisiae*, with structure-activity relationship. 2H4M possessed the highest antifungal activity among test compounds.

### 2.2. Growth Recovery of S. cerevisiae bck1Δ and slt2Δ Mutants by Sorbitol

**Figure 3:**
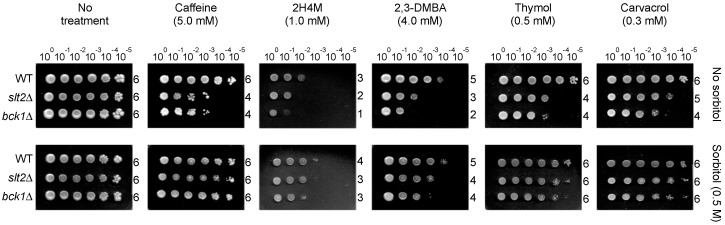
Yeast dilution bioassay showing sensitivity of *S. cerevisiae* *slt2*Δ and *bck1*Δ mutants to caffeine (5 mM; control) and test compounds (2H4M, 2,3-DMBA, thymol, carvacrol) was remediated by sorbitol. Results indicate the test compounds negatively affected cell wall integrity system of fungi.

In sorbitol remediation bioassay, sensitivity of *slt2*Δ and *bck1*Δ to carvacrol, thymol, 2H4M or 2,3-DMBA was alleviated by sorbitol (caffeine: positive control for cell wall perturbation) (See Experimental section and [47] for method). The level of growth of *slt2*Δ and *bck1*Δ on sorbitol-containing media was 10 to 100 times higher compared to controls without sorbitol (Figure 3). Therefore, the remediation by sorbitol indicates that disruption of cell wall integrity in fungi is one contributing mechanism of how the screened benzaldehydes (including thymol and carvacrol) exerted antimycotic activity (alone or in combination with monoterpenoid phenols; See below). 

### 2.3. Chemosensitization Test in S. cerevisiae: Co-Application of Thymol or Carvacrol with 2H4M Augmented the Antifungal Efficacy of Test Compounds in S. cerevisiae

Antifungal chemosensitization was examined based on European Committee on Antimicrobial Susceptibility Testing (EUCAST) broth dilution protocol for yeasts [48], where both minimum inhibitory concentrations (MICs) and minimum fungicidal concentrations (MFCs), and thus, their respective Fractional Inhibitory Concentration Indices (FICI) and Fractional Fungicidal Concentration Indices (FFCI), of *S. cerevisiae* WT, *slt2*Δ and *bck1*Δ were calculated (See Experimental section for calculations and concentrations of test compounds).

**Figure 4:**
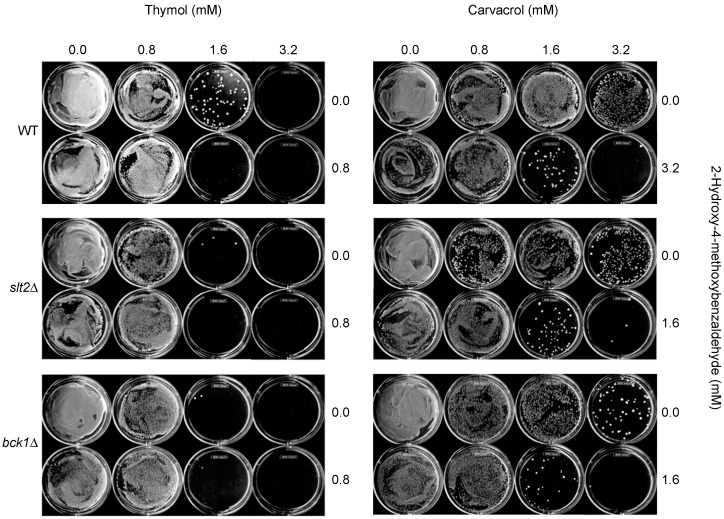
Chemosensitization test in *S. cerevisiae* (thymol or carvacrol + 2H4M). Exemplary plate bioassay showing co-application of thymol or carvacrol (1.6 to 3.2 mM) with 2H4M (0.8 to 3.2 mM) completely inhibited the growth of *S*. *cerevisiae* strains (WT, *slt2*Δ, *bck1*Δ).

For FFCIs of carvacrol, “synergistic” FFCIs (*i.e*., FFCI ≤ 0.5) were not identified between 2H4M and carvacrol for the test strains (Table 4). However, despite the absence of calculated synergism (as determined by “indifferent” interactions [49]) (Table 4), there was increased antifungal activity of 2H4M and carvacrol (*viz*., chemosensitizing effect; FFCIs = 0.6 to 0.8) in WT, *slt2*Δ and *bck1*Δ, which was reflected in lowered MFCs of test compounds when they were co-applied. For example, co-application of 2H4M (3.2, 1.6 or 1.6 mM for WT, *slt2*Δ and *bck1*Δ, respectively) with carvacrol (3.2 mM for all strains) completely prevented the yeast growth (on recovery agar plate)*,* while individual treatment of each compound, alone, at the same dosages allowed the survival of yeast strains (See also Figure 4). Noteworthy is that *slt2*Δ (MAPK) and *bck1*Δ (MAPKKK) required much lower concentration of 2H4M (1.6 mM) compared to WT (3.2 mM) to achieve complete inhibition of yeast growth, thus demonstrating that the knockout strains are less capable of responding to the cell wall perturbation induced by 2H4M, and are therefore more susceptible to the molecule.

For FICIs of carvacrol, although no synergism was found for FICIs (Table 4), there was enhanced antifungal activity of 2H4M and carvacrol for WT (FICIs = 1.0), except *slt2*Δ and *bck1*Δ, for which FICI = 2.0 (neutral interaction). For example, co-application of 2H4M (0.8 mM) with carvacrol (0.4 mM) completely inhibited the growth of WT in liquid culture (microtiter plate)*,* while individual treatment of each compound, alone, at the same concentrations allowed the growth of WT.

For FFCIs of thymol, there was enhanced antifungal activity of 2H4M and thymol for WT (FFCIs = 0.6), except *slt2*Δ and *bck1*Δ, for which FFCI = 2.0 (neutral interaction). For example, co-application of 2H4M (0.8 mM) with thymol (1.6 mM) resulted in complete inhibition of WT growth*,* while individual treatment of each compound, alone, at the same concentrations allowed the survival of the strain.

For FICIs of thymol, despite the absence of calculated synergism, there was increased antifungal activity of 2H4M and thymol (*viz*., chemosensitizing effect; FICIs = 0.8 to 1.0) in WT, *slt2*Δ and *bck1*Δ, which was reflected in lowered MICs of 2H4M and thymol when compounds were co-administered. For example, co-application of 2H4M (0.8 mM for all strains) with thymol (0.4, 0.4, 0.2 mM for WT, *slt2*Δ and *bck1*Δ, respectively) completely inhibited the growth of yeast strains*,* while individual treatment of each compound, alone, at the same concentrations allowed the survival of yeasts (Table 4).

Collectively, 2H4M possessed a chemosensitizing capability to carvacrol or thymol in yeast tests, where MICs and MFCs of test compounds were decreased (*viz*., antifungal potency of test compounds was increased) during chemosensitization. Thymol required much lower concentration (*viz*., 0.2 to 0.4 mM for FICIs; 1.6 mM for FFCI) during chemosensitization compared to carvacrol (*viz*., 0.4 mM for FICIs; 3.2 mM for FFCIs) for complete inhibition of yeast growth, indicating structure-activity relationship also existed in monoterpenoid phenols. The “neutral interaction” determined in *slt2*Δ and *bck1*Δ during chemosensitization (*viz*., FICI_CARVACROL_ or FFCI_THYMOL_ = 2.0) indicated that the MICs of carvacrol for *slt2*Δ and *bck1*Δ (0.4 mM) or MFCs of thymol for the same mutants (1.6 mM) already reached the maximum antimycotic level without chemosensitization, while WT required chemosensitization for the improvement of potency of test compounds. Thus, results further reflect the higher susceptibility of *slt2*Δ and *bck1*Δ mutants to cell wall disrupting reagents (*i.e*., 2H4M, monoterpenoid phenols) compared to WT.

**Table 4:** Antifungal chemosensitization of 2H4M (mM) to carvacrol or thymol (mM), tested against *S. cerevisiae* strains: summary of EUCAST-based microdilution bioassays. ^a^

**Yeast Strains Carvacrol**	**Compounds**	**MIC Alone**	**MIC Combined**	**FICI**	**MFC Alone**	**MFC Combined**	**FFCI**
*S. cerevisiae* WT	Carvacrol	0.8	0.4	1.0	6.4 ^b^	3.2	0.8
	2H4M	1.6	0.8		12.8 ^c^	3.2	
*S. cerevisiae slt2*Δ	Carvacrol	0.4	0.4	2.0	6.4	3.2	0.6
	2H4M	1.6	1.6		12.8	1.6	
*S. cerevisiae bck1*Δ	Carvacrol	0.4	0.4	2.0	6.4	3.2	0.6
	2H4M	0.8	0.8		12.8	1.6	
Mean	Carvacrol	0.5	0.4	1.6	6.4	3.2	0.6
	2H4M	1.3	1.1		12.8	2.1	
*t*-test ^d^	Carvacrol	-	*p* < 0.5	-	-	*p* < 0.005	-
	2H4M	-	*p* < 1.0	-	-	*p* < 0.005	-
**Yeast Strains Thymol**	**Compounds**	**MIC Alone**	**MIC Combined**	**FICI**	**MFC Alone**	**MFC Combined**	**FFCI**
*S. cerevisiae* WT	Thymol	1.6	0.4	0.8	3.2	1.6	0.6
	2H4M	1.6	0.8		12.8	0.8	
*S. cerevisiae slt2*Δ	Thymol	0.8	0.4	1.0	1.6	1.6	2.0
	2H4M	1.6	0.8		12.8	12.8	
*S. cerevisiae bck1*Δ	Thymol	0.8	0.2	0.8	1.6	1.6	2.0
	2H4M	1.6	0.8		12.8	12.8	
Mean	Thymol	1.1	0.3	0.8	2.1	1.6	1.5
	2H4M	1.6	0.8		12.8	8.8	
*t*-test ^d^	Thymol	-	*p* < 0.1	-	-	*p* < 0.5	-
	2H4M	-	*p* = 0.0	-	-	*p* < 0.5	-

^a^ MIC, Minimum inhibitory concentration; MFC, Minimum fungicidal concentration; FICI, Fractional Inhibitory Concentration Indices; FFCI, Fractional Fungicidal Concentration Indices; ^b^ Carvacrol was tested up to 3.2 mM. For calculation purpose, 6.4 mM (doubling of 3.2 mM) was used; ^c^ 2H4M was tested up to 6.4 mM. For calculation purpose, 12.8 mM (doubling of 6.4 mM) was used; ^d^ Student’s *t*-test for paired data (combined, *i.e.*, chemosensitization) was *vs.* mean MIC or MFC of each compound (alone, *i.e.*, no chemosensitization) determined in yeast strains.

### 2.4. Chemosensitization Test in Filamentous Fungi: Co-Application of Thymol or Carvacrol with 2H4M

Antifungal chemosensitization was further explored in filamentous fungal pathogens (*Aspergillus*, *Penicillium*) according to the CLSI microdilution bioassay protocol [44], where both MICs and MFCs, and thus, both FICIs and FFCIs, of fungal pathogens were determined (See Experimental section for concentrations of test compounds).

For FFCIs of carvacrol in *Aspergillus*, “synergistic” FFCI values (*i.e*., FFCI ≤ 0.5) were found between carvacrol and 2H4M for all *Aspergillus* strains examined (FFCIs = 0.3 to 0.4) (Table 5). Prior studies in yeasts showed that mutations in the antioxidant system, such as oxidative MAPK pathway, could result in the development of fungal resistance to cell wall perturbing agents [50,51,52,53]. Therefore, antioxidant mutants of *A. fumigatus* (*sakA*Δ, *mpkC*Δ) were also included in this study to determine whether antioxidant mutants of filamentous fungi develop resistance to 2H4M or monoterpenoid phenols.

**Figure 5:**
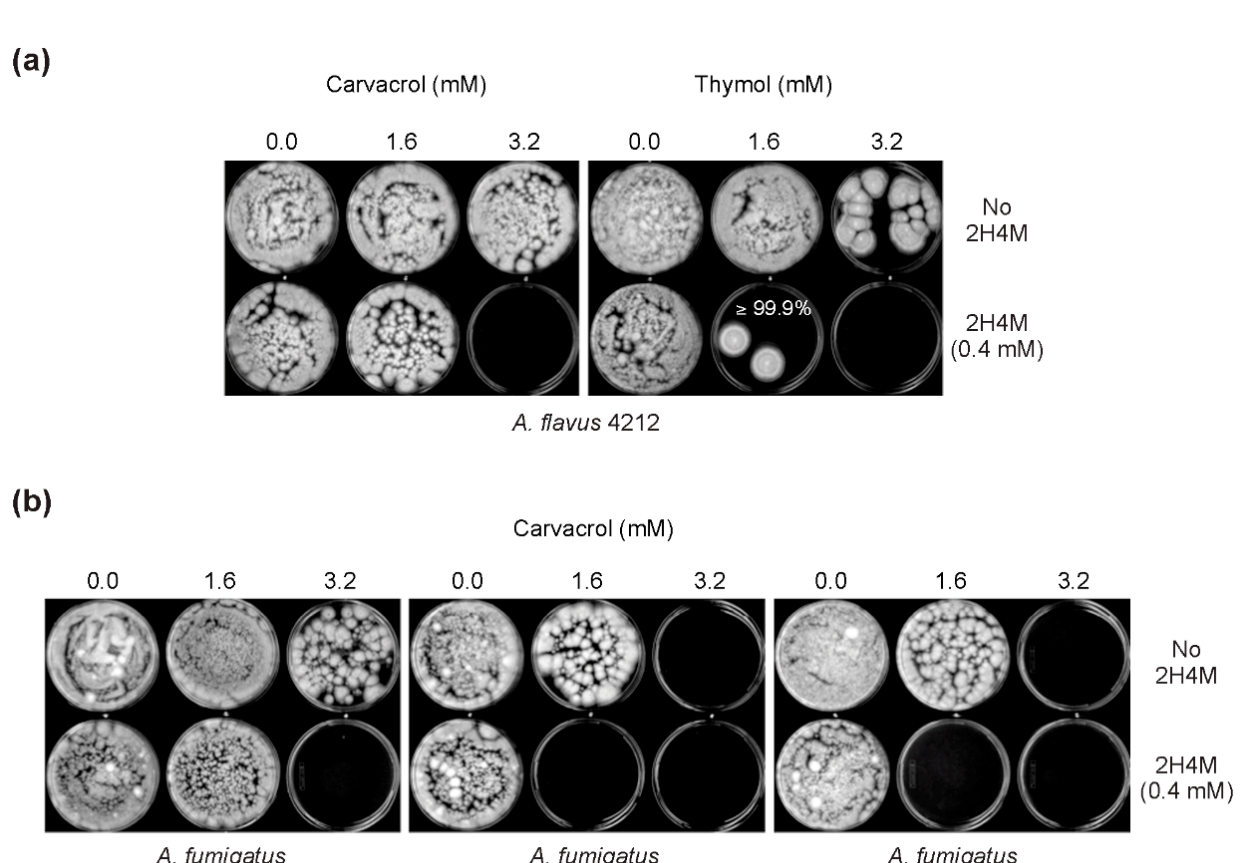
Chemosensitization test in aflatoxigenic *Aspergillus* or *A. fumigatus* (thymol or carvacrol + 2H4M). Exemplary bioassay showing co-application of thymol or carvacrol with 2H4M completely inhibited the growth of (**a**) aflatoxigenic *A*. *flavus* 4212 or (**b**) *A. fumigatus*. Results also showed that oxidative MAPK mutants (*sakA*Δ, *mpkC*Δ) were more sensitive to the chemosensitization compared to WT (Concentrations presented: 1.6 to 3.2 mM, carvacrol or thymol; 0.4 mM, 2H4M).

Results showed that oxidative MAPK mutants (*sakA*Δ, *mpkC*Δ) were more sensitive to carvacrol compared to WT, where the carvacrol values of WT, *sakA*Δ or *mpkC*Δ were (12.8, 3.2, 3.2 mM) for MFC_ALONE_ or (1.6, 0.8, 0.8 mM) for MFC_COMBINED_, respectively. It is surmised that, similar to other phenolics (See Introduction), carvacrol further exacerbated the vulnerability (namely, defects in countering oxidative stress or disruption of cellular redox homeostasis) of the antioxidant mutants (*sakA*Δ and *mpkC*Δ) [27,28] (See also Figure 5). Accordingly, besides the cell wall integrity MAPK system, the operation of the “intact” antioxidant MAPK pathway of *A. fumigatus* should also be important for pathogen’s defense against carvacrol. In *Penicillium*, “synergistic” FFCI values were also determined for all *Penicillium* strains tested (FFCIs = 0.3 to 0.4) (Table 5). Collectively, co-administration of 2H4M with carvacrol lowered the MFCs of either compound (2H4M, carvacrol), which resulted in the achievement of synergism in all *Aspergillus* and *Penicillium* strains tested (Mean FFCI of filamentous fungi = 0.3; Table 5).

**Table 5:** Antifungal chemosensitization of 2H4M (mM) to carvacrol or thymol (mM), tested against *Aspergillus* and *Penicillium* strains: summary of CLSI-based microdilution bioassays. ^a^

**Fungal Strains**	**Compounds**	**MIC Alone**	**MIC Combined**	**FICI**	**MFC Alone**	**MFC Combined**	**FFCI**
*A. fumigatus* AF293	Carvacrol	1.6	0.4	0.8	12.8 ^b^	1.6	**0.3**
	2H4M	0.8	0.4		12.8 ^c^	1.6	
*A. fumigatus sakA*Δ	Carvacrol	1.6	0.4	0.8	3.2	0.8	**0.4**
	2H4M	0.8	0.4		12.8	1.6	
*A. fumigatus mpkC*Δ	Carvacrol	1.6	0.4	0.8	3.2	0.8	**0.4**
	2H4M	0.8	0.4		12.8	1.6	
*A. flavus* 3357	Carvacrol	1.6	0.8	1.0	12.8	1.6	**0.4**
	2H4M	0.8	0.4		3.2	0.8	
*A. flavus* 4212	Carvacrol	1.6	0.8	0.8	12.8	1.6	**0.4**
	2H4M	1.6	0.4		3.2	0.8	
*A. parasiticus* 2999	Carvacrol	1.6	0.8	1.0	12.8	0.8	**0.3**
	2H4M	0.8	0.4		6.4	1.6	
*A. parasiticus* 5862	Carvacrol	1.6	0.8	1.0	12.8	0.8	**0.3**
	2H4M	0.8	0.4		6.4	1.6	
*P. expansum* W1	Carvacrol	0.8	0.2	0.8	12.8	1.6	**0.4**
	2H4M	0.4	0.2		12.8	3.2	
*P. expansum* FR2	Carvacrol	0.8	0.4	1.0	12.8	1.6	**0.3**
	2H4M	0.4	0.2		12.8	1.6	
*P. expansum* W2	Carvacrol	1.6	0.8	1.0	12.8	1.6	**0.3**
	2H4M	0.8	0.4		12.8	1.6	
*P. expansum* FR3	Carvacrol	0.8	0.4	1.0	12.8	1.6	**0.3**
	2H4M	0.8	0.4		12.8	1.6	
Mean	Carvacrol	1.4	0.6	0.9	11.1	1.3	**0.3**
	2H4M	0.8	0.4		9.9	1.6	
*t*-test ^d^	Carvacrol	–	*p* < 0.005	–	–	*p* < 0.005	–
	2H4M	–	*p* < 0.005	–	–	*p* < 0.005	–
**Fungal Strains**	**Compounds**	**MIC Alone**	**MIC Combined**	**FICI**	**MFC Alone**	**MFC Combined**	**FFCI**
*A. fumigatus* AF293	Thymol	1.6	0.4	0.8	3.2	0.8	**0.5**
	2H4M	0.8	0.4		12.8	3.2	
*A. fumigatus sakA*Δ	Thymol	1.6	0.4	0.8	1.6	0.4	**0.5**
	2H4M	0.8	0.4		12.8	3.2	
*A. fumigatus mpkC*Δ	Thymol	1.6	0.4	0.8	1.6	0.4	**0.5**
	2H4M	0.8	0.4		12.8	3.2	
*A. flavus* 3357	Thymol	1.6	0.4	0.8	3.2	1.6	0.6
	2H4M	1.6	0.8		3.2	0.4	
*A. flavus* 4212	Thymol	1.6	0.4	0.8	3.2	1.6	0.6
	2H4M	1.6	0.8		3.2	0.4	
*A. parasiticus* 2999	Thymol	1.6	0.8	1.0	12.8 ^e^	0.8	**0.3**
	2H4M	0.8	0.4		6.4 ^f^	1.6	
*A. parasiticus* 5862	Thymol	1.6	0.8	1.0	12.8	0.8	**0.3**
	2H4M	0.8	0.4		6.4 ^f^	1.6	
*P. expansum* W1	Thymol	0.8	0.4	1.0	12.8	1.6	**0.3**
	2H4M	0.4	0.2		12.8	1.6	
*P. expansum* FR2	Thymol	0.8	0.4	1.0	12.8	1.6	**0.3**
	2H4M	0.4	0.2		12.8	1.6	
*P. expansum* W2	Thymol	1.6	0.8	1.0	12.8	1.6	**0.3**
	2H4M	0.8	0.4		12.8	1.6	
*P. expansum* FR3	Thymol	0.8	0.4	1.0	12.8	1.6	**0.3**
	2H4M	0.8	0.4		12.8	1.6	
Mean	Thymol	1.4	0.5	0.8	8.1	1.2	**0.3**
	2H4M	0.9	0.4		9.9	1.8	
*t*-test	Thymol	–	*p* < 0.005	-	-	*p* < 0.005	-
	2H4M	–	*p* < 0.005	-	-	*p* < 0.005	-

^a^ MIC, Minimum inhibitory concentration; MFC, Minimum fungicidal concentration; FICI, Fractional Inhibitory Concentration Indices; FFCI, Fractional Fungicidal Concentration Indices. Synergistic FICIs and FFCI are in bold; ^b^ Carvacrol was tested up to 6.4 mM. For calculation purpose, 12.8 mM (doubling of 6.4 mM) was used; ^c^ 2H4M was tested up to 6.4 mM. For calculation purpose, 12.8 mM (doubling of 6.4 mM) was used; ^d^ Student’s *t*-test for paired data (combined, *i.e.*, chemosensitization) was *vs.* mean MIC or MFC of each compound (alone, *i.e.*, no chemosensitization) determined in strains; ^e^ Thymol was tested up to 6.4 mM. For calculation purpose, 12.8 mM (doubling of 6.4 mM) was used; ^f^ 99.8% killing.

Regarding FICIs of carvacrol in *Aspergillus*, enhanced activity of 2H4M or carvacrol was also identified during chemosensitization (FICIs = 0.8 to 1.0), although there was no calculated synergism. For example, co-application of 2H4M (0.4 mM) with carvacrol (0.8 mM) achieved complete inhibition of *A. parasiticus* growth*,* while individual treatment of each compound, alone, at the same concentrations resulted in the survival of *A. parasiticus*. However, unlike the MFC testing (See above), the *sakA*Δ and *mpkC*Δ mutants did not show higher sensitivity to carvacrol during MIC testing, when compared to WT (Table 5). In *Penicillium*, despite the no calculated synergism, enhanced activity of 2H4M and carvacrol was also found in all test strains, where FICIs were 0.8 to 1.0. Altogether, co-application of 2H4M and carvacrol resulted in enhancement of antifungal activity of either compound during FICI determination (Mean FICI of filamentous fungi = 0.9; See Table 5).

For FFCIs of thymol in *Aspergillus*, synergistic FFCIs were identified in *A. fumigatus* (FFCI = 0.5) and *A. parasiticus* (FFCI = 0.3). Although no synergism was identified, there was elevated antifungal activity of 2H4M and thymol in *A. flavus* strains (FFCI = 0.6) during chemosensitization (Table 5). As observed in carvacrol test (See above), *A. fumigatus* oxidative MAPK mutants (*sakA*Δ, *mpkC*Δ) were more susceptible to thymol compared to WT, where the thymol values of WT, *sakA*Δ or *mpkC*Δ were (3.2, 1.6, 1.6 mM) for MFC_ALONE_ or (0.8, 0.4, 0.4 mM) for MFC_COMBINED_, respectively. Higher susceptibility to thymol (compared to carvacrol) was also observed in *A. flavus* (3357, 4212) (for example, MIC_COMBINED_ or MFC_ALONE_ of thymol or carvacrol was (0.4 *vs.* 0.8 mM) for MIC_COMBINED_ or (3.2 *vs.* 12.8 mM) for MFC_ALONE_, respectively; Table 5). However, the level of antimycotic activity of carvacrol or thymol was vastly similar in *A. parasiticus* or *P. expansum* strains, except MIC_COMBINED_ of *P. expansum* W1 (*viz*., MIC_COMBINED_ for carvacrol or thymol was 0.2 or 0.4 mM, respectively). In *Penicillium*, “synergistic” FFCI values were also determined in all *Penicillium* strains tested (FFCIs = 0.3) (Table 5). Altogether, co-application of 2H4M and thymol resulted in enhancement of antifungal activity of either compound during FFCI determination (Mean FFCI of filamentous fungi = 0.3; See Table 5). The MFC values of thymol were, in general, lower than that of carvacrol, indicating that, as observed in *S. cerevisiae* (See above), thymol possessed higher antimycotic potency in selected fungi.

Regarding FICIs of thymol in *Aspergillus*, enhanced antimycotic activity of 2H4M or thymol was also identified during chemosensitization (FICIs = 0.8 to 1.0), despite the no calculated synergism. For example, co-application of 2H4M (0.4 mM) with thymol (0.8 mM) resulted in complete inhibition of *A. parasiticus* growth in liquid culture*,* while individual treatment of each compound, alone, at the same concentrations allowed the survival of *A. parasiticus*. However, unlike the MFC testing (See above), the *sakA*Δ and *mpkC*Δ mutants did not show higher sensitivity to thymol compared to WT in MIC testing (Table 5). In *Penicillium*, despite not achieving calculated synergism, enhanced activity of 2H4M and thymol was also identified in all test strains, where FICIs were 1.0. Collectively, co-application of 2H4M and thymol achieved the enhancement of antifungal activity of either compound during FICI determination (Mean FICI of filamentous fungi = 0.8; See Table 5).

### 2.5. Overcoming Fludioxonil Tolerance of A. fumigatus MAPK Mutants by 2H4M Co-Treatment

**Figure 6:**
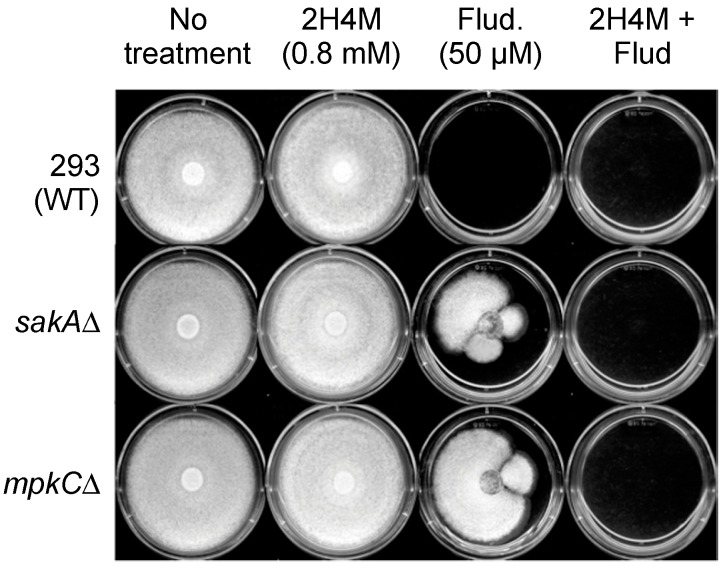
Overcoming fludioxonil tolerance of *A. fumigatus* MAPK mutants (*sakA*Δ, *mpkC*Δ) by 2H4M (agar plate bioassay).

Fludioxonil, a conventional phenylpyrrole fungicide, elicits excessive stimulation of the intact MAPK signaling pathway, which is responsive to high osmotic/oxidative stress [54]. Thus, the immoderate activation of the osmotic/oxidative MAPK signaling system (*HOG1* in *S. cerevisiae*) by fludioxonil results in an energy drain via metabolic shifts from normal growth to exhaustive stress response. Consequently, treatment of fungi with fludioxonil prevents the normal growth of fungal cells. However, fungal strains having mutations in genes of upstream signal transduction pathway, such as osmotic/oxidative MAPK signaling pathway, can evade toxicity exerted by fludioxonil [54]. We demonstrated that *A. fumigatus* MAPK mutants (*sakA*Δ, *mpkC*Δ) exhibited tolerance to 50 μM fludioxonil (thus, were able to develop radial growth on agar), while the growth of WT (AF293) was completely inhibited (Figure 6). However, co-application of sub-fungicidal concentration of 2H4M (at 0.8 mM, where the fungal growth rate was almost not inhibited) with fludioxonil achieved the prevention of fungal tolerance to the fungicide, which resulted in complete growth inhibition of MAPK mutants (*sakA*Δ, *mpkC*Δ) (Figure 6). Cell wall disrupting agents, such as echinocandin drugs, lyse the actively growing hyphal tips of fungi during filamentous fungal growth [23]. It is speculated that the cell wall disturbing capability of 2H4M can enhance the penetration of fludioxonil into the MAPK mutants through perturbed cell wall, which results in complete inhibition of the growth of *A. fumigatus sakA*Δ and *mpkC*Δ on fludioxonil-containing plates.

### 2.6. Antimycotoxigenic Property of 2H4M against A. parasiticus Strains

As mentioned previously (See Introduction), sub-optimal application of fungicide can potentiate the production of mycotoxins in various fungal pathogens. Therefore, the effect of the antimycotic 2H4M, thymol and carvacrol on the production of aflatoxins (AFs) was evaluated in the mycotoxigenic *Aspergillus* strains, *i.e., A. flavus* 3357, 4212 and *A. parasiticus* 2999, 5862. Test compounds were administered at sub-MICs or MFCs (0.125 to 1.000 mM), where the sclerotial development was also monitored.

In *A. flavus*, administration of thymol and carvacrol at 0.125 to 0.500 mM (0.125 to 0.250 mM for 2H4M) enhanced the production of aflatoxins (AFB_1_, AFB_2_; See Figure 1 for structures) compared to control (no treatment) (Table 6). Results indicated that, like other commercial fungicides (See Introduction), carvacrol, thymol or 2H4M could potentiate the biosynthesis of mycotoxins in fungi. However, mycotoxin production was lowered at >0.500 mM of monoterpenoid phenols (compared to no treatment control), while fungal growth (accordingly, mycotoxin production) was completely inhibited at ≥0.500 mM of 2H4M, reflecting higher antifungal potency of 2H4M compared to thymol or carvacrol (There was an observable growth defect across fungal species at 0.5 to 1.0 mM concentrations of each antifungal). 

**Table 6:** Effect of 2H4M and monoterpenoid phenols on the production of aflatoxins and sclerotia. Increased values compared to no treatment control are in bold characters. ^a^

Thymol						
Strains	Concentration	AFB_1_	AFB_2_	AFG_1_	AFG_2_	Sclerotia
*A. flavus* 3357	0.000	5.14 ± 0.87	0.07 ± 0.02	– ^b^	–	6 ± 6
	0.125	**7.38 ± 0.31**	**0.14 ± 0.01**	–	–	**47 ± 5**
	0.250	**7.74 ± 1.16**	**0.14 ± 0.02**	–	–	**84 ± 7**
	0.500	**8.72 ± 0.36**	**0.19 ± 0.01**	–	–	**31 ± 9**
	1.000	1.16 ± 0.48	0.01 ± 0.00	–	–	0 ± 0
*A. flavus* 4212	0.000	4.55 ± 0.08	0.02 ± 0.00	–	–	0 ± 0
	0.125	**5.86 ± 0.15**	**0.03 ± 0.01**	–	–	0 ± 0
	0.250	**6.77 ± 0.38**	**0.05 ± 0.01**	–	–	**3 ± 2**
	0.500	**7.15 ± 0.12**	**0.06 ± 0.01**	–	–	0 ± 0
	1.000	1.56 ± 0.47	0.01 ± 0.00	–	–	0 ± 0
*A. parasiticus* 2999	0.000	14.46 ± 0.31	0.25 ± 0.01	3.07 ± 0.18	0.12 ± 0.01	0 ± 0
	0.125	**18.80 ± 0.06**	**0.50 ± 0.01**	**5.93 ± 0.06**	**0.30 ± 0.01**	**171 ± 23**
	0.250	**23.16 ± 1.26**	**0.71 ± 0.06**	**7.65 ± 0.82**	**0.41 ± 0.04**	**28 ± 13**
	0.500	**18.19 ± 0.50**	**0.49 ± 0.01**	**3.96 ± 0.35**	**0.17 ± 0.03**	0 ± 0
	1.000	0.90 ± 0.44	0.01 ± 0.00	0.02 ± 0.01	0.00 ± 0.00	0 ± 0
*A. parasiticus* 5862	0.000	15.01 ± 0.92	0.27 ± 0.02	3.39 ± 0.24	0.14 ± 0.01	0 ± 0
	0.125	**18.64 ± 1.60**	**0.49 ± 0.06**	**5.98 ± 0.83**	**0.30 ± 0.03**	**166 ± 21**
	0.250	**22.69 ± 1.38**	**0.69 ± 0.07**	**7.54 ± 0.82**	**0.40 ± 0.06**	**17 ± 13**
	0.500	**16.34 ± 1.49**	**0.44 ± 0.05**	3.32 ± 0.48	**0.16 ± 0.04**	0 ± 0
	1.000	0.69 ± 0.22	0.01 ± 0.00	0.01 ± 0.01	0.00 ± 0.00	0 ± 0
**Carvacrol**						
**Strains**	**Concentration**	**AFB_1_**	**AFB_2_**	**AFG_1_**	**AFG_2_**	**Sclerotia**
*A. flavus* 3357	0.000	4.80 ± 0.14	0.06 ± 0.00	– ^b^	–	2 ± 1
	0.125	**5.78 ± 0.31**	**0.09 ± 0.01**	–	–	**64 ± 11**
	0.250	**5.73 ± 0.53**	**0.10 ± 0.01**	–	–	**59 ± 8**
	0.500	**5.96 ± 0.36**	**0.13 ± 0.01**	–	–	0 ± 0
	1.000	1.23 ± 0.45	0.01 ± 0.01	–	–	0 ± 0
*A. flavus* 4212	0.000	5.51 ± 0.56	0.03 ± 0.01	–	–	0 ± 0
	0.125	**6.84 ± 0.66**	**0.05 ± 0.01**	–	–	**1 ± 2**
	0.250	**7.50 ± 0.66**	**0.07 ± 0.01**	–	–	**8 ± 4**
	0.500	**6.50 ± 0.29**	**0.07 ± 0.01**	–	–	0 ± 0
	1.000	0.91 ± 0.32	0.01 ± 0.00	–	–	0 ± 0
*A. parasiticus* 2999	0.000	14.02 ± 1.58	0.25 ± 0.06	2.75 ± 0.54	0.12 ± 0.03	1 ± 1
	0.125	**17.30 ± 0.83**	**0.37 ± 0.03**	**3.22 ± 0.27**	**0.14 ± 0.01**	**62 ± 4**
	0.250	**17.38 ± 0.26**	**0.43 ± 0.01**	**2.90 ± 0.12**	**0.13 ± 0.01**	**46 ± 5**
	0.500	**19.54 ± 0.88**	**0.52 ± 0.04**	2.20 ± 0.13	0.08 ± 0.00	0 ± 0
	1.000	0.09 ± 0.04	0.00 ± 0.00	0.00 ± 0.00	0.00 ± 0.00	0 ± 0
*A. parasiticus* 5862	0.000	13.92 ± 0.36	0.23 ± 0.02	2.84 ± 0.10	0.10 ± 0.02	1 ± 1
	0.125	**16.65 ± 0.88**	**0.35 ± 0.03**	**3.19 ± 0.28**	**0.12 ± 0.01**	**44 ± 6**
	0.250	**16.51 ± 1.07**	**0.39 ± 0.04**	2.68 ± 0.22	**0.11 ± 0.01**	**25 ± 3**
	0.500	**20.10 ± 0.49**	**0.52 ± 0.03**	2.32 ± 0.10	0.07 ± 0.01	0 ± 0
	1.000	0.12 ± 0.12	0.00 ± 0.00	0.00 ± 0.00	0.00 ± 0.00	0 ± 0
**2H4M**						
**Strains**	**Concentration**	**AFB_1_**	**AFB_2_**	**AFG_1_**	**AFG_2_**	**Sclerotia**
*A. flavus* 3357	0.000	4.73 ± 0.23	0.06 ± 0.01	– ^b^	–	4 ± 3
	0.125	**5.03 ± 0.35**	**0.08 ± 0.01**	–	–	0 ± 0
	0.250	**4.84 ± 0.06**	**0.10 ± 0.01**	–	–	0 ± 0
	0.500	NG ^c^	NG	NG	NG	NG
	1.000	NG	NG	NG	NG	NG
*A. flavus* 4212	0.000	4.72 ± 0.24	0.02 ± 0.01	–	–	0 ± 0
	0.125	**6.58 ± 1.02**	**0.05 ± 0.01**	–	–	0 ± 0
	0.250	**5.69 ± 0.55**	**0.04 ± 0.01**	–	–	0 ± 0
	0.500	NG	NG	NG	NG	NG
	1.000	NG	NG	NG	NG	NG
*A. parasiticus* 2999	0.000	15.92 ± 1.28	0.27 ± 0.04	3.20 ± 0.33	0.13 ± 0.03	0 ± 1
	0.125	12.80 ± 0.26	0.24 ± 0.01	2.62 ± 0.08	0.12 ± 0.00	0 ± 0
	0.250	10.16 ± 0.79	0.17 ± 0.02	2.23 ± 0.24	0.06 ± 0.02	0 ± 0
	0.500	NG	NG	NG	NG	NG
	1.000	NG	NG	NG	NG	NG
*A. parasiticus* 5862	0.000	14.21 ± 1.02	0.23 ± 0.03	2.91 ± 0.35	0.11 ± 0.01	0 ± 1
	0.125	12.65 ± 0.53	0.23 ± 0.02	2.65 ± 0.07	0.10 ± 0.01	0 ± 0
	0.250	10.95 ± 1.19	0.19 ± 0.04	2.38 ± 0.43	0.06 ± 0.02	0 ± 0
	0.500	NG	NG	NG	NG	NG
	1.000	NG	NG	NG	NG	NG

^a^ Thymol, Carvacrol, 2H4M: mM; aflatoxins: μg/cm^2^; Sclerotia: numbers per plate; ^b^ Below the detection limit; ^c^ NG, No growth (accordingly, no aflatoxin detected).

In *A. parasiticus*, treatment of fungi with thymol and carvacrol at 0.125 to 0.500 mM also enhanced the production of aflatoxins (AFB_1_, AFB_2_, AFG_1_, AFG_2_; See Figure 1 for structures) compared to control (no treatment) (Table 6). As observed in *A. flavus*, mycotoxin production was lowered at >0.500 mM of monoterpenoid phenols (compared to no treatment control), while fungal growth (accordingly, mycotoxin production) was also completely prevented at ≥0.500 mM of 2H4M. In general, *A. parasiticus* produced higher amounts of aflatoxins compared to *A. flavus*, when treated with thymol or carvacrol (See Table 6).

Notable observation is that, unlike the *A. flavus* strains, 2H4M actually inhibited the aflatoxin production in *A. parasiticus* at all test concentrations (Table 6), where the level of inhibition was commensurate with the 2H4M concentration. Therefore, results strongly indicated that “strain specificity” also existed for the “antimycotoxigenic activity” of 2H4M.

Of note, in a prior study, oxidative stress regulated the sclerotial differentiation, where application of antioxidant modulators of reactive oxygen species prohibited the development of sclerotia [55]. Furthermore, aflatoxin biosynthesis and sclerotial differentiation were co-modulated by oxidative stress [55]. Therefore, sclerotial development/differentiation is considered as the indicator of fungal stress response, especially to oxidative stress.

In this study, treatment of *A. flavus* or *A. parasiticus* strains with thymol or carvacrol also enhanced the sclerotial differentiation, depending on dosages and types of test compounds. *A. flavus* 3357 produced higher number of sclerotia compared to *A. flavus* 4212 (with both thymol and carvacrol), while *A. parasiticus* strains developed more sclerotia with thymol than with carvacrol (Table 6). Conversely, sclerotia development was almost not detected with the treatment of 2H4M, strongly indicating that: (1) the mechanism of action of 2H4M for the antifungal or antimycotoxigenic activity against *Aspergillus* strains is considered to be different from that of monoterpenoid phenols; and (2) antifungal or antimycotoxigenic activity is dependent upon types (or species) of *Aspergillus* strains examined. Noteworthy is the effect of cell wall stress in the production of the secondary metabolite pyomelanin in *A. fumigatus.* Previous study showed that pyomelanin production was enhanced when constitutive cell wall stress was applied to *A. fumigatus* [56], strongly indicating the link between secondary metabolite production and cell wall stress. Recent study further revealed that genes involved in the pyomelanin synthesis were all up-regulated when the cell wall integrity MAPK mutant Δ*mpkA* was cultured under cell wall stress [57].

The strain specificity for the differential antimycotoxigenic activity with 2H4M (*i.e*., *A. flavus vs. A. parasiticus*), as determined in this study, is a new observation, which may reflect the different level of cell wall stress triggered by 2H4M in pathogens, depending on types of fungal species. Precise determination of the mechanism of differential activity regarding the antifungal or antimycotoxigenic activity of 2H4M warrants future study.

## 3. Experimental Section

### 3.1. Fungal Strains and Culture Conditions

Fungal strains used in this study are summarized in Table 2. *Aspergillus* and *Penicillium* strains were grown at 35 °C and 28 °C (Forma Scientific, Marietta, OH, USA), respectively, on potato dextrose agar (PDA). *Saccharomyces cerevisiae* wild type (WT) BY4741 (*Mat* a *his3*Δ*1 leu2*Δ*0* *met15*Δ*0* *ura3*Δ*0*) and selected single gene deletion mutants (*bck1*Δ, *slt2*Δ) were procured from Invitrogen (Carlsbad, CA, USA) and Open Biosystems (Huntsville, AL, USA; See also *Saccharomyces* Genome Database [30]). Yeast strains were cultured on Synthetic Glucose (SG; Yeast nitrogen base without amino acids 0.67%, glucose 2% with appropriate supplements: uracil 0.02 mg/mL, amino acids 0.03 mg/mL) or Yeast Peptone Dextrose (YPD; Bacto yeast extract 1%, Bacto peptone 2%, glucose 2%) medium at 30 °C. All chemicals for culturing fungi were procured from Sigma Co. (St. Louis, MO, USA).

### 3.2. Chemicals

Benzaldehyde (basal structure) and its structural analogs (*i.e*., fourteen benzaldehyde derivatives; See Figure 1), two monoterpenoid phenols (thymol, carvacrol; cell wall integrity disruptors), caffeine, sorbitol, and fludioxonil (fungicide) were procured from Sigma Co. (St. Louis, MO, USA). Each compound was dissolved in dimethylsulfoxide (DMSO from AMRESCO (Solon, OH, USA); absolute DMSO amount: <2% in media) before incorporation into culture media (except for those plates used in aflatoxin assays; see below). In all tests, control plates (*i.e*., No treatment) contained DMSO at levels equivalent to that of cohorts receiving antifungal agents, within the same set of experiments (See Tables and Figures).

### 3.3. Susceptibility Testing

#### 3.3.1. Agar Plate Bioassay in *S. cerevisiae*

Petri plate-based yeast dilution bioassays were performed on the WT and mutants (*slt2*Δ, *bck1*Δ) to assess effects of screened compounds on the cell wall integrity system. Yeast strains were exposed to 1 to 5 mM of benzaldehyde analogs screened. 1 × 10^6^ cells of the WT or cell wall integrity mutants (*bck1*Δ, *slt2*Δ) of *S. cerevisiae*, cultured on YPD plate, were serially diluted 10-fold in SG liquid medium supplemented with amino acids and uracil (See above) five times to yield cell dilution cohorts of 10^6^, 10^5^, 10^4^, 10^3^, 10^2^ and 10^1^ cells. Cells from each dilution of respective strains were spotted on SG agar incorporated with individual compounds, such as benzaldehyde analogs or other test reagents. Yeast cells were incubated at 30 °C. Results were monitored/evaluated based on a designated value of the highest dilution where colonies became visible after 5 to 7 days of incubation, as follows: Score “0”—no colonies were visible from any of the dilutions, Score “1”—only a colony from the spot with the undiluted cells (10^6^ cells), Score “2” only colonies from the spots with the undiluted (10^6^) and 10^5^ cells were visible, *etc.*, while Score “6”—colonies were visible from all dilution spots. Therefore, each unit (1 to 6) of numerical difference was equivalent to a 10-fold difference in the sensitivity of the yeast strain to the treatment.

#### 3.3.2. Agar Plate Bioassay in *Aspergillus*: Overcoming Fludioxonil Resistance of *A. fumigatus sakA*Δ and *mpkC*Δ Mutants

Measurement of overcoming fungal (*A. fumigatus sakA*Δ, *mpkC*Δ) tolerance to fludioxonil was based on comparison of radial growth between treated and control fungal colonies (See Figure 6). For the above assays, fungal conidia (5 × 10^3^) were diluted in phosphate buffered saline and applied as a drop onto the center of PDA plates containing: (1) No treatment (control); (2) 2H4M (0.1, 0.2, 0.3, 0.4, 0.5, 0.6, 0.7, 0.8, 0.9, 1.0 mM); (3) fludioxonil (50 μM); and (4) 2H4M + fludioxonil. Growth was observed for five to seven days at 35 °C.

#### 3.3.3. Liquid Bioassay in Filamentous Fungi (CLSI) and *S. cerevisiae* (EUCAST)

To determine the precise level of compound interaction between 2H4M (0.1, 0.2, 0.4, 0.8, 1.6, 3.2, 6.4 mM) and monoterpenoid phenols (0.1, 0.2, 0.4, 0.8, 1.6, 3.2, 6.4 mM) in the strains of filamentous fungi (*Aspergillus*, *Penicillium*), checkerboard bioassays (triplicate) (0.4 × 10^4^–5 × 10^4^ CFU/mL) were performed in microtiter wells using a broth microdilution method (in RPMI 1640 medium; Sigma Co., St. Louis, MO, USA), according to protocols outlined by the Clinical and Laboratory Standards Institute (CLSI) M38-A [44]. RPMI 1640 medium was supplemented with 0.03% l-glutamine and buffered with 0.165 mM 3-(*N*-morpholino) propanesulfonic acid (Sigma Co., St. Louis, MO, USA). Minimum Inhibitory Concentrations (MICs), lowest concentration of agents showing no visible fungal growth in microtiter wells (200 μL per well), were assessed after 48 h. Minimum Fungicidal Concentrations (MFCs), lowest concentration of agents achieving ≥99.9% fungal death, were determined following completion of MIC assays by spreading entire volumes of microtiter wells (200 μL) onto individual PDA (recovery) plates, and culturing for additional 48 h (at 28 or 35 °C, depending on types of filamentous fungi). Compound interactions, *i.e.*, Fractional Inhibitory Concentration Indices (FICIs) and Fractional Fungicidal Concentration Indices (FFCI), were calculated as follows: FICI or FFCI = (MIC or MFC of compound A in combination with compound B/MIC or MFC of compound A, alone) + (MIC or MFC of compound B in combination with compound A/MIC or MFC of compound B, alone). Levels and types of compound interactions between antifungal agents (2H4M and thymol or carvacrol) were defined as: synergistic (FICI or FFCI ≤ 0.5) or indifferent (FICI or FFCI > 0.5–4) [49]. 

Compound interaction between 2H4M (0.1, 0.2, 0.4, 0.8, 1.6, 3.2, 6.4 mM) and thymol or carvacrol (0.1, 0.2, 0.4, 0.8, 1.6, 3.2 mM) in *S. cerevisiae* was also determined by using checkerboard broth dilution bioassays in microtiter plates (with SG liquid medium; Sigma Co., St. Louis, MO, USA) according to methods outlined by the European Committee on Antimicrobial Susceptibility Testing (EUCAST) [48].

### 3.4. Growth Recovery Bioassay for S. cerevisiae bck1Δ and slt2Δ Mutants

To examine the effect of antifungal agents (2-Hydroxy-4-methoxybenzaldehyde (2H4M), 2,3-Dimethoxybenzaldehyde (2,3-DMBA), thymol, carvacrol) on cell wall integrity system of fungi, sorbitol recovery bioassays were performed (See [47] for method). Tenfold serially diluted (See “Agar plate bioassays in *S. cerevisiae*” above) strains of *S. cerevisiae* BY4741, *bck1*Δ and *slt2*Δ were spotted on (1) SG only (No treatment control); (2) SG + caffeine (5.0 mM; Positive control), thymol or carvacrol (0.1 0.2, 0.3, 0.4, 0.5 mM), 2H4M (0.1, 0.2, 0.3, 0.4, 0.5, 0.6, 0.7, 0.8, 0.9, 1.0, 1.1, 1.5 mM) or 2,3-DMBA (0.5, 1.0, 2.0, 3.0, 4.0, 5.0 mM) (*viz*., Testing sensitivity of *bck1*Δ and *slt2*Δ mutants to test compounds); and (3) SG + sorbitol (0.5 M) + caffeine, thymol, carvacrol, 2H4M or 2,3-DMBA (*viz*., Testing recovery of *bck1*Δ and *slt2*Δ mutants, by sorbitol, from sensitivity to antifungal reagents). Cell growth was monitored for 5 to 7 days. If the growth score of *S. cerevisiae bck1*Δ and *slt2*Δ on the sorbitol-containing medium was higher than that on the “no sorbitol” medium, the test compounds were considered to affect cell wall integrity system.

### 3.5. Aflatoxin Analysis of Fungal Cultures

Test compounds (thymol, carvacrol, 2H4M), dissolved in distilled water, were filter-sterilized (Millipore Millex GP, Billerica, MA, USA) before incorporation into PDA. Ten mL of PDA containing test compounds (See Table 6 and text for concentrations) were poured into Petri dish (60 mm) in triplicate for each concentration. Fungal spores of *A. parasiticus* and *A. flavus* strains, stored on PDA at 30 °C for 7 days, were collected on a sterile cotton swab and dispersed in 0.05% Tween 80 solution (Sigma Co., St. Louis, MO, USA). Fungal conidia (200 CFU/5 µL) from the prepared suspensions were applied as a drop onto the center of PDA plates, and were incubated at 30 °C for 5 days. The entire culture (agar medium plus fungal mat) was then extracted in 50 mL methanol (Fisher, Thermo Fisher Scientific, Waltham, MA, USA), and 1 mL aliquot was filtered through a 0.45 µm nylon syringe filter (Pall Acrodisc, Port Washington, NY, USA). An aliquot of 20 µL was analyzed for aflatoxins using an Agilent 1100 HPLC system (Santa Clara, CA, USA), which consists of a degasser, autosampler, quaternary pump, fluorescence detector, and a postcolumn photochemical reactor for enhanced detection (PHRED, Aura Industries, New York, NY, USA). Aflatoxins were separated on an Inertsil 4.6 mm × 250 mm ODS-3 column (GL Sciences, Torrance, CA, USA) using an isocratic mobile phase of water/acetonitrile/methanol (45/25/30) at a flow rate of 1 mL/min with fluorescence detection (365 nm excitation, 455 nm emission). Aflatoxin values were determined using standard curves prepared for each of the individual compounds.

### 3.6. Statistical Analysis

Statistical analysis (student’s *t*-test) was performed based on “Statistics to use” [58], where *p* < 0.05 was considered significant.

## 4. Conclusions

In this study, levels of compound interactions between monoterpenoid phenols (carvacrol, thymol) and a chemosensitizer (2H4M) were determined for the enhancement of antifungal efficacy. Key features identified for the antifungal or antimycotoxigenic potential of compounds are as follows: (1) Among fifteen benzaldehyde analogs examined, nine compounds inhibited the growth of *bck1*Δ and *slt2*Δ, the cell wall integrity mutants of *S. cerevisiae*, with structure-activity relationship. 2H4M exhibited the highest antifungal activity among test compounds; (2) 2H4M possessed a chemosensitizing capability to carvacrol or thymol in yeasts, where chemosensitization enhanced the antimycotic potency of test compounds. The 2H4M, a cell wall perturbing chemosensitizer, and monoterpenoid phenols could affect common cellular targets, *i.e.*, cell wall integrity system of fungi, which results in synergistic inhibition of fungal growth; (3) In yeast chemosensitization, thymol required much lower concentration to achieve complete inhibition of yeast growth compared to carvacrol, thus reflecting structure-activity relationship; (4) In filamentous fungal tests, co-administration of 2H4M with carvacrol or thymol resulted in the achievement of synergism in *Aspergillus* and *Penicillium* strains; (5) Carvacrol or thymol further exacerbated the vulnerability (namely, defects in countering oxidative stress) of the oxidative MAPK mutants (*sakA*Δ, *mpkC*Δ) of *A. fumigatus*; (6) *A. fumigatus* or *A. flavus* were more susceptible to thymol compared to carvacrol, while the level of antimycotic activity of carvacrol or thymol was vastly similar in *A. parasiticus* or *P. expansum* (compound-strain relationship); (7) Co-application of 2H4M with fludioxonil overcame fungal tolerance to fludioxonil, where the cell wall interfering capability of 2H4M might enhance the susceptibility of the oxidative MAPK mutants (*A. fumigatus sakA*Δ, *mpkC*Δ) to fludioxonil, possibly by increased penetration of fludioxonil into the fungal cell through perturbed cell wall; (8) Thymol and carvacrol enhanced aflatoxin production in *A. flavus* and *A. parasiticus*. Although 2H4M also potentiated the aflatoxin production in *A. flavus*, this compound reduced the aflatoxin production in *A. parasiticus* at all concentrations examined (*i.e*., strain specificity for the antimycotoxigenic activity of 2H4M); (9) Thymol or carvacrol also enhanced the sclerotial differentiation in *A. flavus* or *A. parasiticus*, depending on dosages and types of test compounds. *A. flavus* 3357 produced higher number of sclerotia compared to *A. flavus* 4212 (w/carvacrol or thymol), while *A. parasiticus* developed more sclerotia with thymol than with carvacrol. However, sclerotia development was almost not detected with the treatment of 2H4M.

In previous studies in yeasts, another signaling pathway, namely the “intact” oxidative MAPK pathway, was also shown to be important for fungal susceptibility to cell wall perturbing agents [50,51,52,53]. Results showed that mutations in the antioxidant system could develop fungal resistance to cell wall disrupting agents. For example, the *S. cerevisiae* oxidative MAPK pathway mutants (e.g., MAPK or MAPK kinase mutants, the upstream transmembrane osmosensor or histidine kinase osmosensor mutants in the same signaling cascade, *etc.*) exhibited tolerance to the cell wall-perturbing agents, such as calcofluor white [51,52,53]. Therefore, existence of the “intact” oxidative MAPK pathway of fungi is necessary for effective control of pathogens. Nonetheless, the antioxidant mutants of *A. fumigatus* (*sakA*Δ, *mpkC*Δ) tested in this study did not develop tolerance to 2H4M or monoterpenoid phenols, while 2H4M further overcame fungal tolerance to the phenylpyrrole fungicide fludioxonil.

In conclusion, 2H4M, a natural phenolic compound, possesses a potential to serve as an antimycotic chemosensitizer in combination with monoterpenoid phenols. 2H4M-mediated chemosensitization, as described in this study, can modulate/debilitate the cell wall integrity system of fungal strains, which can lower effective doses of antimycotic agent co-administered. Future studies are needed for comprehensive determination of optimum chemosensitization in various fungal pathogens by including additional cell wall disrupting agents. It would also be interesting to know whether antifungal activity correlates with chemosensitizing activity, or whether a circumstance similar to that for piperazinyl quinolone with fluconazole [32], exists for benzaldehyde analogs with no antifungal activity.
